# Sub-galeal abscess: A rare sequel of an infected scalp sebaceous cyst

**DOI:** 10.1016/j.ijscr.2020.09.063

**Published:** 2020-09-24

**Authors:** Mamoun Nabri, Mohammed Alharbi, Amnah Al-Sayyid, Kawthar Alabdrabalrasol, Khairi Hassan, Hussam Al-Jehani

**Affiliations:** a Department of Surgery, King Fahad Hospital of University, Imam Abdulrahman Bin Faisal University, Dammam, Saudi Arabia; b Medical Intern, King Fahad Hospital of University, Imam Abdulrahman Bin Faisal University, Dammam, Saudi Arabia; c Medical Student in Imam Abdulrahman Bin Faisal University, King Fahad Hospital of University, Imam Abdulrahman Bin Faisal University, Dammam, Saudi Arabia; d Department of Neurosurgery, King Fahad Hospital of University, Imam Abdulrahman Bin Faisal University, Dammam, Saudi Arabia

**Keywords:** Subgaleal, Abscess, Sebaceous, Cyst, Vacuum, Dressing

## Abstract

**Introduction:**

Subgaleal abscess is a rare condition in which suppuration under the galea aponeurotica (epicranial aponeurosis) occurs. Diagnosis of subgaleal abscess is guided by high index of suspicion, imaging of the head by computed topography will confirm the diagnosis and detect osteomyelitis or intracranial extension.

**Presentation of case:**

We describe a case of extensive subgaleal abscess that presented as an infected scalp sebaceous cyst, in male diabetic patient, its management and the role of vaccum assisted closure (V.A.C.) dressing which was never described in such atypical presentation.

**Discussion:**

The subgaleal space is the between the galea aponeurotica and periosteum of the cranial bones, subgaleal abscess may result from hematogenous infection or contiguous spread, and the diagnosis may not be initially obvious^4^ The predominant organism isolated from post-traumatic and post-surgical scalp infections is *Staphylococcus aureus* (Haines and Chou, 1985). Diagnosis and management will be guided by head CT scan, management of subgaleal abscess should focus on effective intravenous antibiotic therapy, immediate surgical drainage of abscess and debridement of necrotic tissue.

**Conclusion:**

Subgaleal abscess is a rare complication, imaging can prevent serious complications. Negative pressure wound therapy is an invaluable tool for closure of the wounds.

## Highlights

• Subgaleal abscess with skull osteomyelitis is a rare.
• Diabetic patient with progressive whole scalp swelling and headache for 2 weeks, associated with fever.
• High suspicion will help in diagnosis, CT scan will confirm the diagnosis, detect osteomyelitis or intracranial extension.
• Vacuum assisted closure dressing, worked dwell for us, post drainage and debridement which never describe in this condition.

## 1 Introduction

The galea aponerutica (epicranial aponeurosis) is a thin tendentious connective tissue connecting the two bellies of the occipitofrontalis muscle, the periosteum of the cranial bones is lying under galeal layer, the subgaleal space is potential space for collection of blood or infective material [1]. Abscess formation under the galeal layer is usually due to iatrogenic causes (e.g. neurosurgical procedures, fetal scalp monitoring), or due to penetrating scalp or head injury [2]. A common scenario is extension of infection from nearby structures, scalp skin is in proximity to the galea, superficial skin infections can invade the galea and may present as a subgaleal abscess [[2], [3], [4], [5], [6]].

The work has been reported in line with the SCARE criteria and cite the following paper [12].

## 2 Case report

A 53 years old diabetic man presented with progressive scalp swelling and headache for 2 weeks, associated with fever. Upon examination the swelling is located at the vertex of skull, with an opening with purulent discharge with cellulitis around the lesion. On examination the swelling is doughy in consistency with no fluctuation, cystic lesion is felt in the middle of the wound. The patient underwent excision of the infected cyst, debridement and drainage of an abscess. Tissue pathology revealed an infected sebaceous cyst and Staphylococcus Aureus was cultured. During post-operative period he improved slightly and cellulitis subsided.

**Image 1:**
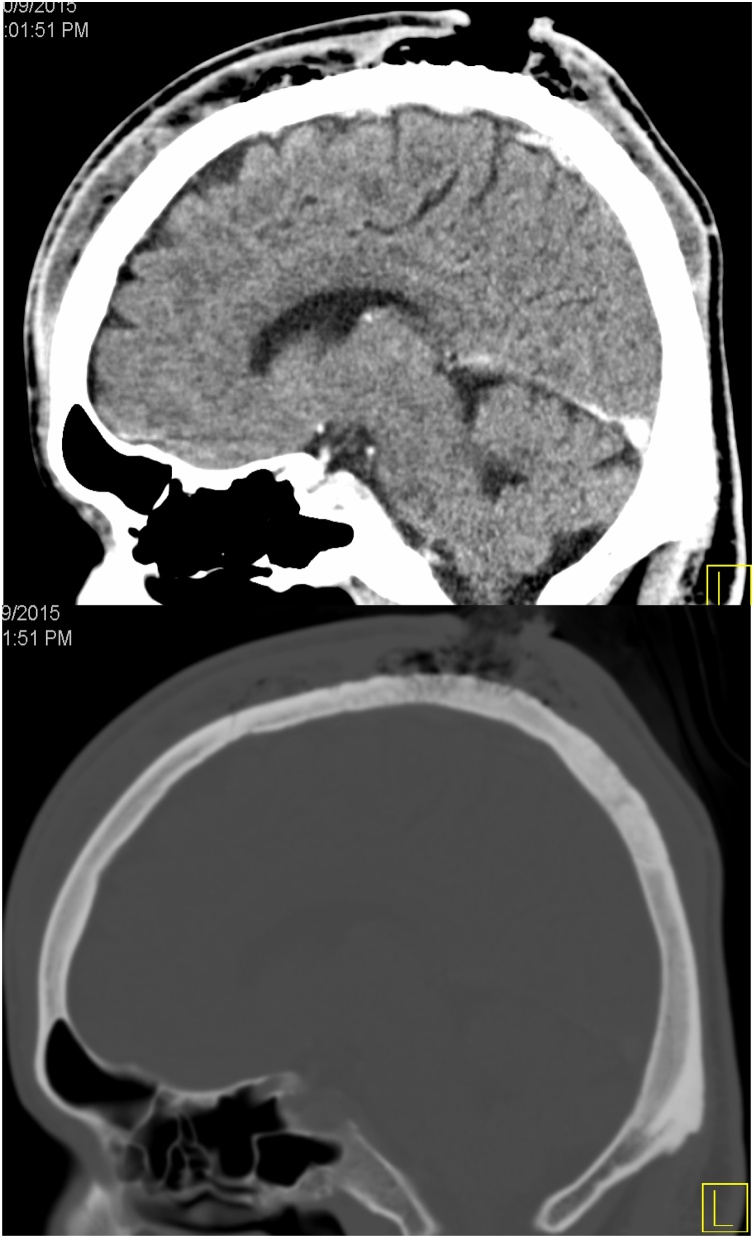
Head CT showing subgaleal collection, the bony wound shows involvement of cranial bones.

**Image 2:**
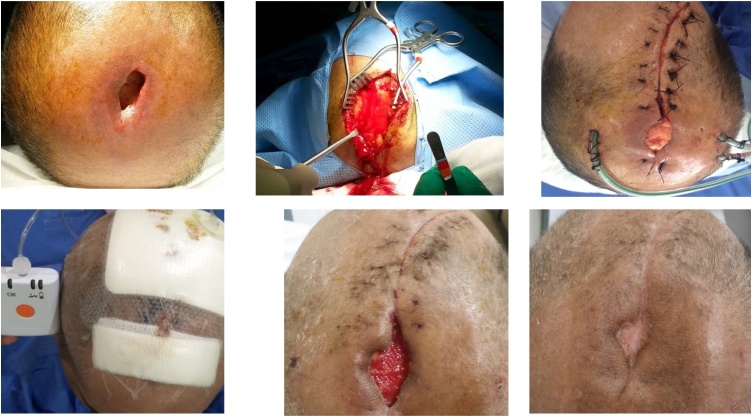
**From left up:** Pre-operative, Aggressive debridement, Post-operative day one. **From left down:** Portable negative pressure wound therapy, Post-operative day 18, at one month follow up.

On post-operative day 3, the wound was still discharging copious amount of pus though the patient was not febrile. A computed scan of the head revealed subgaleal collection with air pockets and evidence of outer plate of skull bone osteomyelitis (Image 1). Patient was taken for surgical debridement of the infected galeal tissue, copious amount of pus was drained. The outer plate of skull bone was debrided. Two drains placed in the pocket above the eyebrows anteriorly and one in the occipital pocket posteriorly. Negative pressure wound therapy (V.A.C) was used on the first day post-op that aided in resolution of the infection. The patient was continued on intravenous antibiotics. The patient had an uneventful recovery and was discharged home after 12 days. On follow up the skin defect healed completely and the patient didn’t need any skin flap (Image 2).

## 3 Discussion

The subgaleal space is the between the galea aponeurotica and periosteum of the cranial bones, it is a potential space.

The subgaleal space is a very extensive area spanning all of the cranial convexity. It is delimited circumferentially by the attachments of the galea aponeurotica: anteriorly to the bony orbital ridge bilaterally; laterally to the zygomatic arch and the auricular muscles on each side; and posteriorly to the nuchal line [7].

Diagnosis of abscess formation in this space is usually related to postoperative complications and encountered in some operative procedure to the head, ears or sinuses. Infection can extended from other close foci, in this case the subagaleal collection can be missed if not suspected. Serious complications can arise if subgaleal involvement is not diagnosed early, skull bone osteomylitis and intracranial extension are difficult clinical scenarios.

However, subgaleal abscess may result from hematogenous infection or contiguous spread, and the diagnosis may not be initially obvious [4].

The predominant organism isolated from post traumatic and post surgical scalp infections is *Staphylococcus aureus* [8]. However, other organisms such as Strep pyogenes [9] and *Eikenella corrodens* [5] have been reported. In the neonatal age group, in which abscess formation can be a complication of fetal scalp electrode monitoring, *Staphylococcus epidermidis* accounted for 58% of positive cultures in one review [10]. Polymicrobial infections may also occur with the presence of anaerobes in association with either Staph aureus or Strep pyogenes [2].

Head CT is often needed in the diagnosis of subgaleal abscess, and if operative debridement is not promptly performed, subgaleal abscess may further progress to life-threatening septicemia, osteomyelitis, and even subdural or brain abscess or meningitis [11].

Immunocompromised patients such as diabetics are prone to recurrent infections especially in the skin; managing such infections early and aggressively reduces the risk of extension of these infections to adjacent potential spaces. They should have an urgent brain imaging and urgent management.

Management of subgaleal abscess should focus on effective intravenous antibiotic therapy, immediate surgical drainage of abscess and debridement of necrotic tissue. Culture of the infected tissue to identify the causative organism is extremely important with the emergence of resistant microorganisms.

Finally, we experienced an impressive result using the negative pressure wound therapy (V.A.C). This type of dressing uses a vacuum machine to apply a negative pressure of -80 to -120 to the wound. It removes all the liquid or semi-liquid infective or necrotic material from the wound and improves vascularity to the tissue, which in result aid in regeneration of healthy granulation tissue. Our patient had the advantage of reusable pocket size VAC therapy and was discharged on repeated changes in the dressing clinic every three days for five weeks. The wound improved dramatically and the skin defect healed by secondary intention.

## 4 Conclusion

Subgaleal abscess is a rare complication of infected scalp skin cysts. Early diagnosis and imaging can prevent serious complications, osteomyelitis, and even subdural or brain abscess. Negative pressure wound therapy is an invaluable tool for closure of infected scalp wounds and will help in tissue regeneration and infection clearance.

## Declaration of Competing Interest

No potential conflict of interest relevant to this article was reported.

## Funding

There are no sources of funding for this research.

## Ethical approval

Not applicable. The study is exempt from ethical approval in our institution.

## Consent

Written informed consent was obtained from the patient for publication of this case report and accompanying images. A copy of the written consent is submitted

No identifying details have been used in the article.

## Author contribution

Dr. Mamoun A. Nabri, Dr. Mohammed Alharbi contributed to the paper by collecting all important data and information pertaining to the case.

Dr. Amnah Al-Sayyid, Dr. Hussam Al-Jehani and Dr Khairi Hassan contributed to the paper by reviewing the final manuscript.

Kawthar Alabdrabalrasol contributed to the paper by reviewing all the available literature related to the case.

## Registration of research studies

Not applicable.

## Guarantor

Dr. Mamoun A. Nabri.

## Provenance and peer review

None funded, externally peer-reviewed.
